# Macrophage Reprogramming: Emerging Molecular Therapeutic Strategies for Nephrolithiasis

**DOI:** 10.3390/biom15081090

**Published:** 2025-07-28

**Authors:** Meng Shu, Yiying Jia, Shuwei Zhang, Bangyu Zou, Zhaoxin Ying, Xu Gao, Ziyu Fang, Xiaofeng Gao

**Affiliations:** 1 Department of Urology, Changhai Hospital of Shanghai, Naval Medical University, Shanghai 200433, China; shumeng06@smmu.edu.cn (M.S.); jia_yiying@smmu.edu.cn (Y.J.); zhangshuwei@smmu.edu.cn (S.Z.); bangyu_zou@163.com (B.Z.); yingzhaoxin1996@163.com (Z.Y.); gaoxu.changhai@foxmail.com (X.G.); 2 Shanghai Key Laboratory of Cell Engineering, Shanghai 200433, China

**Keywords:** nephrolithiasis, macrophage polarization, CaOx, cellular reprogramming, precision medicine

## Abstract

Nephrolithiasis, predominantly driven by calcium oxalate (CaOx) crystal deposition, poses a significant global health burden due to its high prevalence and recurrence rates and limited preventive/therapeutic options. Recent research has underscored a pivotal role for macrophage polarization in nephrolithiasis pathogenesis. Pro-inflammatory phenotype macrophages exacerbate crystal-induced injury and foster stone formation by amplifying crystal adhesion via an NF-κB–IL-1β positive-feedback axis that sustains ROS generation and NLRP3 inflammasome activation, whereas anti-inflammatory phenotype macrophages facilitate crystal clearance and tissue repair. We have summarized the research on treating nephrolithiasis and related renal injury by targeting macrophage polarization in recent years, including therapeutic approaches through pharmacological methods, epigenetic regulation, and advanced biomaterials. At the same time, we have critically evaluated the novel therapeutic strategies for macrophage reprogramming and explored the future development directions of targeting macrophage reprogramming for nephrolithiasis treatment, such as using single-cell/spatial omics to reveal the heterogeneity of macrophages in the stone microenvironment, chimeric antigen receptor macrophages (CAR-Ms) as a potential therapy for specific crystal phagocytosis in certain areas, and multi-omics integration to address inter-patient immune differences. This review highlights that macrophage reprogramming is a transformative frontier in nephrolithiasis management and underscores the need for further research to translate these molecular insights into effective clinical applications.

## 1. Introduction

Nephrolithiasis is one of the most common urological disorders worldwide, imposing a significant burden on global health [1]. CaOx stones are the most common type of nephrolithiasis, accounting for over 80% of cases [2]. From January 2011 to November 2019, the prevalence of nephrolithiasis has shown a statistically significant increase in the Medicare population, rising from 1.84% to 2.34% (*p* < 0.0001) in the USA [3]. Similarly, the prevalence of nephrolithiasis in China is also notably high, with 1 out of every 17 adults being troubled by nephrolithiasis [4]. A variety of demographic factors, such as age, gender, and race, have been shown to significantly influence the occurrence of CaOx stones [5]. Furthermore, nephrolithiasis is typically a disease with a high recurrence rate and lifelong persistence, with a 10-year recurrence rate of 52% [4]. However, the exact causes of nephrolithiasis formation remain elusive, and there is a lack of effective drugs for its treatment and prevention. Currently, minimally invasive surgery is the primary treatment for nephrolithiasis patients [6], and there is an urgent need for new management strategies. Current treatment options for nephrolithiasis mainly include conservative treatment and surgical treatment, which have many limitations, such as side effects, ineffectiveness in some patients, and high recurrence rates [5]. These limitations highlight the necessity of preventive measures and innovative strategies.

In recent years, studies have shown that the immune microenvironment has emerged as a critical regulator of nephrolithiasis formation, with local immune responses, inflammatory cell infiltration, cytokine release, and immune cell polarization collectively influencing stone pathogenesis [7]. In a cross-sectional study, researchers identified that a higher immune-inflammatory index (SII) level was independently associated with nephrolithiasis in people younger than 50 years old [8], highlighting the link between immune activation and nephrolithiasis risk. The activation of immune cell infiltration not only initiates local inflammation but also exacerbates tissue damage via cytokine/chemokine release. For example, Tarek M. El-Achkar et al. conducted a spatial transcriptomics analysis and found significant immune damage and matrix remodeling in the renal papillary tissues of nephrolithiasis patients. This immune-mediated damage may create a favorable microenvironment for stone attachment and growth [9]. Specifically, CaOx crystals further drive renal injury by inducing oxidative stress, ROS production, and inflammasome activation (e.g., NLRP3), leading to IL-1β release and sustained inflammation [10,11]. Similarly, the production of reactive oxygen species (ROS), activation of inflammasomes, and increased expression of molecules in the inflammatory cascade are associated with the deposition of crystals in the kidneys [12]. In this crystal–inflammation–crystal reaction process, macrophages actively participate by releasing a variety of cytokines that are crucial for the inflammatory immune response [13]. Additionally, oxalate can induce mitochondrial dysfunction, disrupt the redox homeostasis of monocytes, and further exacerbate oxidative stress [14]. This continuous inflammatory stimulation promotes crystal attachment and changes in the local matrix environment, providing favorable conditions for stone formation [15].

Thus, the infiltration of immune cells and inflammatory responses play a crucial role in promoting nephrolithiasis formation. In view of the complexity of immune cell infiltration and inflammatory response, macrophage polarization is the core regulatory mechanism, and its dynamic balance has a decisive influence on the formation and regression of nephrolithiasis. The specific mechanism will be detailed below.

## 2. Macrophage Polarization: A Key Mechanism in the Nephrolithiasis

### 2.1. Phenotypic Characteristics of Macrophages

Macrophages are crucial immune cells that play a central role in maintaining tissue homeostasis and eliminating pathogens [16]. Macrophage polarization, particularly the M1 and M2 phenotypes, is critically involved in regulating immune responses [17,18]. M1 and M2 macrophages exhibit different characteristics in terms of surface markers, metabolic features, and functional roles [19]. M1 macrophages, stimulated by IFN-γ and LPS, express surface markers like CD86 and CD68, secrete pro-inflammatory cytokines (IL-1β, IL-6, TNF-α), rely on glycolysis for energy, and promote inflammation and tissue damage via pro-inflammatory factors and ROS. In contrast, M2 macrophages, induced by IL-4 and IL-13, express markers such as CD206 and arginase-1, secrete anti-inflammatory cytokines (IL-10, TGF-β), utilize oxidative phosphorylation, and function to inhibit inflammation, promote tissue remodeling, angiogenesis, and repair [20,21,22,23,24,25].

However, this binary classification is increasingly recognized as an oversimplification. Emerging evidence, particularly from single-cell RNA sequencing (scRNA-seq) studies, reveals that macrophages exhibit remarkable phenotypic plasticity and heterogeneity in response to microenvironmental cues, rather than fitting strictly into M1 or M2 categories [26,27]. Therefore, a more comprehensive classification system based on pro-inflammatory and anti-inflammatory functions may be more appropriate.

### 2.2. Macrophage Activation and Polarization in Nephrolithiasis

The pathological progression of nephrolithiasis is closely related to the activation and polarization of macrophages [28]. In hyperoxaluric conditions, renal tubular epithelial cells injured by CaOx crystals release damage-associated molecular patterns (DAMPs) and chemokines (e.g., MCP-1, IL-8), which recruit circulating monocytes to the site of injury [29,30]. Once at the injury site, these circulating monocytes differentiate into macrophages to clear the crystals. However, when the crystal load exceeds the macrophages’ processing capacity, the crystals cannot be cleared in a timely manner, potentially leading to a local pro-inflammatory response [31,32]. In a pro-inflammatory environment, macrophages further release pro-inflammatory cytokines, such as IL-1β and IL-8, which, by amplifying the immune response, may reinforce the negative effects on crystal formation, thereby accelerating crystal-to-stone transformation [33]. In this context, macrophages can polarize into either the pro-inflammatory type or the anti-inflammatory type, which inhibits inflammation. Meanwhile, studies have shown that the proportion of M1-type macrophages is significantly increased in the papillary region of the kidneys of stone-forming patients compared to those without stones. This increase is further aggravated by the accumulation of mineral deposits. On the other hand, if there is an accumulation of anti-inflammatory factors in the local environment of the renal papilla, such as increased levels of IL-10 and TGF-β, macrophages may polarize into the M2 type. M2 macrophages then exhibit functions of inhibiting the inflammatory response and promoting tissue repair, which may contribute to the dissolution and natural expulsion of stones to some extent [34].

### 2.3. The Balance of Pro-/Anti-Inflammatory Polarization of Macrophages Plays a Key Role in Nephrolithiasis

**Figure 1:**
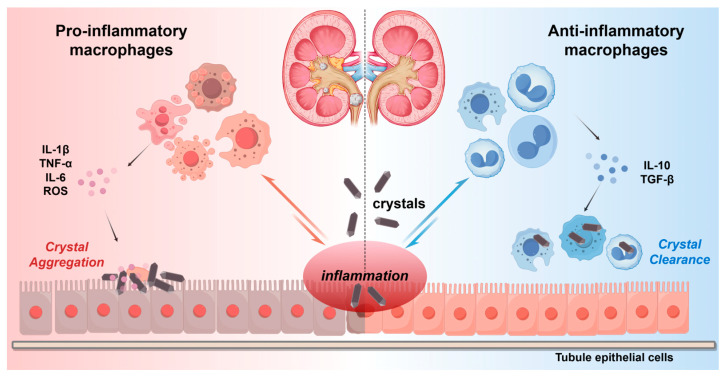
Macrophage polarization in nephrolithiasis. Different polarization states of macrophages play different roles in nephrolithiasis. Pro-inflammatory macrophages can intensify crystal deposition and inflammatory responses by secreting pro-inflammatory cytokines (IL-1β, TNF-α, IL-6) and reactive oxygen species (ROS). Anti-inflammatory macrophages promote tissue repair and crystal clearance through anti-inflammatory cytokines (IL-10, TGF-β).

The balance between pro-inflammatory and anti-inflammatory (based on M1/M2) macrophages affects the progression of CaOx disease [21,35]. Figure 1 illustrates the different roles of different polarization states of macrophages in nephrolithiasis. In the early stage of nephrolithiasis, crystals such as CaOx are released by damaging the renal tubular epithelial cells, activating local macrophages to polarize towards the pro-inflammatory type [36,37]. Pro-inflammatory macrophages release pro-inflammatory factors such as TNF-α and IL-1β through the TLR/NF-κB signaling pathway, intensifying the inflammatory microenvironment and promoting crystal adhesion and aggregation [33]. Animal models have demonstrated that the high expression of M1 markers iNOS and CD86 is positively correlated with nephrolithiasis formation [38]. Additionally, the reactive oxygen species (ROS) produced by M1-type macrophages can further damage the basement membrane of renal tubules, providing sites for crystal attachment; M2-type macrophages can suppress the inflammatory response by secreting IL-10 and TGF-β and enhance phagocytic function to clear microcrystals and prevent the formation of stone cores [13,39]. In summary, the pro-inflammatory and anti-inflammatory polarization balance of macrophages acts as a “double-edged sword” in nephrolithiasis. It regulates inflammation, crystal clearance, and tissue repair, ultimately affecting the development of nephrolithiasis.

## 3. Molecular Therapeutic Translation Potential for Macrophage Reprogramming in Nephrolithiasis

With advancing research, macrophage-reprogramming strategies have emerged as a focal point in preclinical nephrolithiasis studies. In this section, we critically synthesize and categorize the latest cell-based and rodent model investigations that aim to manipulate macrophage polarization for attenuating crystal deposition and renal injury. Notably, we explicitly emphasize the current lack of clinical trials and large-animal validation in this field.

### 3.1. Direct Regulatory Strategies Targeting Macrophage Polarization

**Figure 2:**
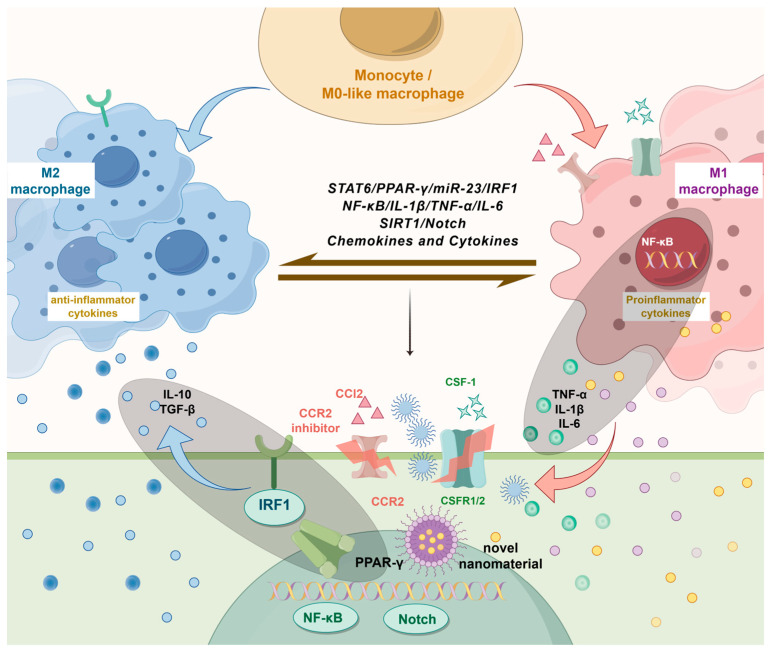
Therapeutic strategies targeting macrophage polarization in nephrolithiasis. Various strategies for the treatment of nephrolithiasis targeting macrophage polarization have been reported recently. They include direct regulation of cytokines and chemokines (e.g., CCR2 inhibitors, CSF-1), targeting key signaling pathways (e.g., PPAR-γ, NF-κB, Notch), and epigenetic reprogramming. Novel biomaterials and nanomaterials are also shown as emerging approaches for modulating macrophage behavior. These strategies aim to shift macrophage polarization from the pro-inflammatory state to the anti-inflammatory state, reducing inflammation and promoting tissue repair in nephrolithiasis.

Macrophages exhibit high plasticity, enabling them to polarize into either the pro-inflammatory or anti-inflammatory phenotype in response to specific stimuli [40,41]. During the pathological progression of urinary stones, the polarization state of macrophages significantly influences the formation and development of these stones [13,33]. By modulating specific molecular pathways, cytokines, and chemokines, therapeutic strategies can shift the balance between pro-inflammatory and anti-inflammatory macrophages, potentially altering the course of nephrolithiasis. Figure 2 illustrates how these therapeutic strategies can reduce inflammation and promote tissue repair by shifting macrophage polarization from a pro-inflammatory phenotype to an anti-inflammatory phenotype.

#### 3.1.1. Direct Regulation of Targeted Chemokine Network and Cytokines

Table 1 summarizes key findings from recent studies on macrophage polarization regulation via chemokine/cytokine signaling and critical pathways, as discussed in Section 3.1.1 and Section 3.1.2.

**Chemokines:** Chemokines, a class of low-molecular-weight proteins, orchestrate immune cell trafficking through gradient-mediated chemotaxis, playing pivotal roles in inflammatory localization [49]. Within the pathological progression of urolithiasis, the chemokine network exerts dual effects: it recruits immune cells (e.g., macrophages, neutrophils) to sites of renal parenchyma and urinary tract inflammation, augmenting local defense mechanisms while simultaneously amplifying the cytokine milieu in inflamed regions. This dynamic process exacerbates tissue damage and microenvironmental alterations critical for stone formation [50]. Cytokines similarly regulate lithogenic pathways. Pro-inflammatory cytokines such as interleukin-1β (IL-1β), interleukin-6 (IL-6), and tumor necrosis factor-α (TNF-α) amplify injury responses by promoting inflammation and augmenting the activation of immune effector cells [51]. Epidemiological studies demonstrate that elevated serum levels of these cytokines in nephrolithiasis patients correlate with chronic renal fibrosis and inflammatory cell infiltration [52].

**CCL2-CCR2 regulatory axis:** The chemokine CCL2 (formerly termed monocyte chemoattractant protein–1, MCP-1) orchestrates the trafficking of monocytes, macrophages, and diverse immune cells (e.g., T cells, dendritic cells) to inflammatory foci via binding to its cognate receptor CCR2, thereby facilitating immune cell infiltration in pathological contexts [53]. CCR2 is a monocyte chemotactic factor associated with oxidative stress and inflammation [54]. However, MCP-1 is widely recognized as a central mediator of inflammatory responses in nephrolithiasis pathogenesis, particularly through its role in recruiting monocytes and macrophages to promote crystal-induced renal injury. The CCL2-CCR2 signaling axis plays a key role in promoting pathological angiogenesis, tumor cell survival and invasion, and the recruitment of immunosuppressive cells [55].

Emerging preclinical evidence demonstrates that pharmacological CCR2 antagonism attenuates CaOx crystal-induced M1 polarization in THP-1 monocytes/macrophages by suppressing TNF-α, IL-6, and iNOS expression. This mechanism subsequently ameliorates CaOx-triggered oxidative stress, inflammatory cascades, and apoptotic signaling in human renal tubular epithelial cells (HK-2). In glyoxylic acid-induced nephrolithiasis murine models, CCR2 inhibitors significantly reduce tubular epithelial injury and crystal deposition, likely through disrupting the CCL2/CCR2-axis-mediated cross-talk between crystal-laden tubular cells and pro-inflammatory macrophages [42]. These findings suggest that the CCL2-CCR2 regulatory axis may serve as a novel therapeutic target for counteracting CaOx crystal-induced oxidative stress and inflammatory responses through suppression of macrophage M1 polarization.

**CSF-1:** Macrophage colony-stimulating factor (CSF1) is a major growth factor required to control the differentiation, survival, proliferation, and renewal of monocytes and macrophages, which sustains macrophage polarization towards an M2-like phenotype [56,57]. Studies have widely recognized that the CSF1/CSF1R and CSF2/CSF2R signaling pathways play crucial roles in macrophage polarization [58]. In the field of renal calculi, Taguchi et al. demonstrated that CaOx crystal deposition was markedly elevated in the kidneys of CSF-1-deficient mice compared to wild-type mice. Administration of recombinant human CSF-1 not only upregulated the expression of M2 macrophage-related genes but also significantly decreased the number of kidney crystals in both CSF-1-deficient and wild-type mice [43].

**IL-10:** IL-10 is an important anti-inflammatory cytokine that can activate the STAT3 signaling pathway by binding to the IL-10 receptor on the surface of macrophages, thereby promoting the polarization of macrophages to the M2 type [59]. In a 2019 study, IL-10, along with IL-4, IL-1a, GM-CSF, and IL-1β, was identified as a urinary inflammation-related factor that could accurately distinguish between controls and patients with urolithiasis [60]. In addition, in a 2023 study, researchers analyzed circulating monocyte RNA collected before and after a high-oxalate (8 mmol) diet intervention in three healthy subjects and found that IL-10 may play an important role in oxalate-mediated cellular changes. Exogenous IL-10 treatment can reverse the oxalate-induced M1 polarization of macrophages and promote the transformation of macrophages into M2 type, thereby inhibiting crystal deposition and inflammatory response [44].

#### 3.1.2. Targeting Key Signaling Pathways

In addition to molecular pathway targeting, epigenetic regulation provides a new intervention dimension for macrophage phenotype switching.

**PPAR-γ-associated signaling pathways:** The PPAR (Peroxisome Proliferator-Activated Receptor) signaling pathway is a nuclear-receptor-mediated transcriptional regulatory pathway primarily involved in biological processes such as lipid metabolism, inflammatory responses, and cellular differentiation [61]. PPAR-γ signaling plays a pivotal role in the regulation of macrophage functions [62]. A study in a murine renal crystal deposition model revealed that pioglitazone mediates the shift from M1-dominant to M2-dominant macrophages under crystal stimulation through the PPAR-γ/*miR-23* axis. Specifically, pioglitazone binds to PPAR-γ, which subsequently interacts with the promoter region of *pre-miR-23* to enhance *miR-23* expression in macrophages. The upregulated *miR-23* then targets interferon regulatory factor 1 (IRF1) and Pknox1, thereby suppressing M1 macrophage polarization and attenuating renal crystal deposition and inflammatory injury [45]. Another separate study demonstrated that *STAT6* facilitates renal fibrosis by suppressing fatty acid oxidation and promoting macrophage-to-myofibroblast transition (MMT) through its interaction with PPARα. In a murine model of CaOx crystal-induced nephropathy, administration of the *STAT6* inhibitor AS1517499 significantly attenuated both renal injury and fibrotic progression [63]. These preclinical research findings suggest that targeting PPAR-γ-associated signaling pathways may become a potential molecular biological target for the treatment of nephrolithiasis by regulating macrophage polarization.

**NF-κB–NLRP3–IL-1β feed-forward cascade:** Within the microenvironment of calcium oxalate nephrolithiasis, upon uptake of crystals by macrophages, an NADPH oxidase-dependent ROS surge is triggered, which in turn promotes NLRP3 inflammasome assembly and activates caspase-1, leading to the rapid release of mature IL-1β. IL-1β, in turn, can activate the IKK–NF-κB signaling pathway, transactivating the transcription of NLRP3 and pro-IL-1β and also driving the expression of pro-inflammatory macrophage genes such as iNOS, TNF-α, and IL-6, locking macrophages in a state of sustained pro-inflammatory polarization [64]. The resulting NF-κB–NLRP3–IL-1β positive feedback loop, on the one hand, directly damages the renal tubular epithelium through ROS and pyroptosis, exposing adhesive niches; on the other hand, by means of the upregulation of VCAM-1, MCP-1, and OPN, it provides an “anchoring soil” for crystal deposition and aggregation, facilitating the continuous growth of stones [65]. Notably, a 2023 study employing both in vivo and in vitro experimental models demonstrated that *FKBP5* deficiency suppresses macrophage M1 polarization and chemotaxis via inhibition of NF-κB signaling transduction. This regulatory mechanism concurrently attenuates crystal–cell adhesion phenomena, ultimately mitigating renal crystal aggregation and ameliorating tubular epithelial injury in murine models of CaOx nephrolithiasis [46]. In addition, deficiency or pharmacological inhibition of NLRP3 (e.g., by MCC950) can induce the conversion of infiltrated macrophages from the pro-inflammatory phenotype to the anti-inflammatory phenotype (CD45^+^F4/80^+^CD11b^+^CD206^+^TGF-β^+^), accompanied by attenuated fibrosis and reduced crystal deposition [47].

**Notch signaling pathway:** Previous studies have demonstrated that the Notch signaling pathway modulates the activation, infiltration, and phenotypic switching of various immune cells, including macrophages and T lymphocytes [66]. Notably, Notch signaling plays a pivotal role in macrophage differentiation and polarization [67]. In 2023, researchers observed that Sirt1 overexpression in macrophages promoted an M2-polarized phenotype, which subsequently reduced apoptosis in co-cultured TCMK1 renal epithelial cells. Further in vivo validation revealed that tail vein infusion of SIRT1-overexpressing macrophages (mimicking M2 macrophage therapy) significantly attenuated CaOx crystal deposition and renal injury in murine models. Mechanistically, this therapeutic effect was mediated through regulatory interactions with the Notch signaling pathway [48].

#### 3.1.3. Epigenetic Reprogramming

Advances in epigenetics have increasingly clarified its regulatory mechanisms in human disease pathogenesis. Zeng et al. demonstrated that acetate treatment upregulated histone H3 acetylation in renal tubular cells, particularly at the H3K9 and H3K27 promoter regions, while simultaneously inducing the expression of *microRNA-130a-3p*, *-148b-3p*, and *-374b-5p*, ultimately resulting in the suppression of CaOx crystal formation [68]. Ye et al. identified that Lgals3 (a β-galactoside-binding lectin) binds directly to the key glycolytic enzyme pyruvate kinase M2 (PKM2), inhibiting its ubiquitination while simultaneously enhancing histone lactylation of H3K18, ultimately facilitating CaOx stone formation and renal injury [69]. Furthermore, epigenetic regulation dynamically modulates macrophage activation and polarization in response to local environmental signals, playing a critical role in immune responses and emerging as a novel strategy for targeting the immunological microenvironment in nephrolithiasis [70,71]. This process involves mechanisms such as DNA methylation, histone modification, chromatin remodeling, and non-coding RNA-mediated regulation, which alter gene expression patterns without modifying the underlying DNA sequence [72]. These mechanisms enable precise control of macrophage responsiveness to environmental stimuli. In this section, we focus on the critical roles of histone modification and DNA methylation in macrophage reprogramming. Table 2 summarizes the latest advances in epigenetic regulation of macrophage polarization, including histone modifications and DNA methylation.

**Histone modifications:** Histone modifications play a critical regulatory role by modulating chromatin structure and transcriptional accessibility. These modifications are covalent post-translational modifications of residues located in the tails of histone proteins and are catalyzed by specific enzymes, such as methyltransferases, demethylases, acetyltransferases, and deacetylases [79,80]. There are several types of covalent modifications, including acetylation, methylation, ubiquitination, SUMOylation, phosphorylation, ADP-ribosylation, and emerging histone modifications such as lactylation, citrullination, and crotonylation [81].

Histone methylation, as a reversible and dynamic chromatin modification, is tightly regulated through post-translational modifications of histone tails. This process involves three key components: methyltransferases (“writers”) that dynamically add methyl groups, demethylases (“erasers”) that specifically remove them, and effector proteins (“readers”) that recognize the modified states to mediate downstream biological effects [82].

Typically, monomethylation of histones such as H3K27, H3K9, H4K20, H3K79, and H2BK5 is associated with gene activation. In contrast, trimethylation of histone H3 lysine 4 (H3K4me3) is closely linked to active gene transcription, transcriptional pausing release, and elongation. Trimethylation of H3K9, H3K27, and H3K79, however, is associated with transcriptional repression [70,83]. In 2019, it was discovered that the histone methyltransferase Setdb2 suppresses transcription by trimethylating histone 3 (H3K9me3) at NF-κB binding sites on the promoters of inflammatory cytokine genes, thereby regulating the shift of macrophages from an inflammatory phenotype to a repair phenotype [73]. In 2022, research found that the methyltransferase DOT1L directly controls lipid gene synthesis in macrophages through methylation of H3K79 on Srebf1/2 (sterol regulatory element-binding transcription factors 1/2). In in vivo experiments, inhibition or deletion of DOT1L leads to hyperactivation of macrophages, which may be associated with a pro-inflammatory phenotype (M1 type) [74]. The histone demethylase JMJD3 can specifically remove trimethylation of lysine 27 on methylated histone H3 (H3K27me3). Studies have found that type I interferon (IFNβ) regulates polarization towards an anti-inflammatory phenotype (M2 type) by reducing the α-ketoglutarate/succinate ratio and blocking the JMJD3-IRF4-dependent pathway [84].

The acetylation status of macrophage histones directly impacts inflammatory cytokine production, tissue repair capacity, and immunomodulatory function [85]. Histone acetyltransferases (HATs) and histone deacetylases (HDACs) regulate histone acetylation levels and gene transcription through a balanced mechanism [86]. Growing evidence suggests that inhibiting HDACs can reduce macrophage M1 polarization, thereby alleviating CaOx crystal-induced renal injury. In a mouse model of oxalate nephropathy, injecting animal chitosan siRNA nanoparticles (NPs) targeting histone deacetylase 5 (HDAC5) into macrophages to inhibit its expression regulated KLF2 and NALP3, improving renal function, reducing inflammation, decreasing neutrophil accumulation, and mitigating tubular injury [75]. In acute kidney injury, the histone deacetylase (HDAC) inhibitor trichostatin A (TSA) can enhance autophagy and promote the M2 macrophage phenotype [87]. Moreover, the NAD-dependent deacetylases, the sirtuin (SIRT) protein family, play a significant role in macrophage polarization and reprogramming. Studies have shown that SIRT1, SIRT2, SIRT3, SIRT5, and SIRT6 can all promote the polarization of macrophages towards the M2 phenotype [76,88,89]. SIRT1 achieves this by inhibiting the NOTCH signaling pathway, while SIRT3 does so by deacetylating FOXO1. In stone-induced mouse models, intravenous infusion of macrophages overexpressing SIRT1/3 significantly reduced the deposition of CaOx crystals and renal injury [90].

In recent years, more histone modifications have been recognized. In 2018, a study on rhabdomyolysis-induced acute kidney injury found that hemoglobin released from necrotic muscle cells during rhabdomyolysis activates platelets, increasing ROS production and histone citrullination in macrophages. This process enhances the production of macrophage extracellular traps (METs) and exacerbates renal injury [91]. In 2019, researchers proposed that M1 macrophages have an endogenous “lactate clock.” In the late stage of M1 macrophage polarization, histone acetylation, regulated by this clock, increases the expression of homeostatic genes involved in wound healing (e.g., Arg-1), leading to a shift towards M2-type characteristics [92]. In 2024, it was found that ACSS2 inhibitors can reduce the expression of IL-1β and subsequent IL-1β-dependent macrophage activation by lowering the level of histone H3 lysine 9 crotonylation (H3K9cr), thereby delaying tubular cell senescence and the progression of fibrosis [77].

These findings highlight the therapeutic potential of drugs targeting histone modification pathways. As research on epigenetic regulators gains more attention, histone modification drugs are emerging as promising candidates for developing novel anti-histone therapies by manipulating macrophage phenotypes.

**DNA methylation:** DNA methylation is a fundamental epigenetic mechanism controlling macrophage development and functional plasticity. This covalent modification primarily occurs at CpG islands in gene promoters, regulating gene expression by inhibiting transcription [93]. DNA methylation is catalyzed by DNA methyltransferases (DNMTs), including DNMT1, DNMT3a, and DNMT3b, and can be removed by the ten-eleven translocation (TET) family of methylcytosine dioxygenases, which include TET1, TET2, TET3, and TET4 [94]. Studies have shown that DNMT3b regulates macrophage polarization by binding to the methylation region of PPARγ1. Knocking out DNMT3b in vitro promotes the expression of Arg-1 and increases M2 macrophage polarization [95,96]. Furthermore, recent studies using high-throughput sequencing and mass spectrometry (MS) have analyzed the exosomal proteomics and DNA methylation data in blood samples from individuals with and without nephrolithiasis. These studies found that overexpressed proteins and hypomethylated genes in nephrolithiasis samples are associated with immune responses [97]. Additionally, the DNA methyltransferase inhibitor 5-aza-2′-deoxycytidine has been shown to rescue ATP1A1 inhibition induced by crystal deposition. This intervention alleviates multiple pathological processes, including oxidative stress, inflammatory cascades, apoptosis, crystal adhesion, and subsequent stone formation [78]. Notably, while these findings establish DNA methylation as a key epigenetic regulator in stone formation, the specific mechanistic relationship between DNA methylation patterns and macrophage polarization in the renal stone microenvironment remains incompletely characterized. Further research is needed to clarify whether DNA methylation directly controls macrophage phenotypic switching and how these epigenetic modifications interact with other regulatory pathways in stone pathogenesis.

### 3.2. Anti-Inflammatory and Antioxidant Treatments Indirectly Regulate Macrophage Polarization

Table 3 compiles key research findings on anti-inflammatory and antioxidant therapies, nanotechnology-based approaches, and biomaterials, as well as gene editing and cellular therapy strategies.

#### 3.2.1. Anti-Inflammatory Drugs Derived from Natural Products

Sulforaphane (SFN), a natural compound abundant in cruciferous vegetables, exhibits potent anti-inflammatory and antioxidant properties. Experimental studies utilizing a murine model of CaOx nephrocalcinosis demonstrated that SFN significantly suppresses TLR4 and IRF1 expression while attenuating M1-polarized macrophage activation induced by CaOx monohydrate (COM)-stimulated supernatants from renal tubular epithelial cells (TECs). Mechanistically, SFN enhances Nrf2-mediated transcriptional activation of *miR-93-5p*, which directly targets and downregulates TLR4 and IRF1 mRNA [98]. Parallel mechanistic insights were observed in acute kidney injury (AKI) research involving puerarin, an isoflavone derivative and the primary bioactive constituent of the traditional Chinese herb Pueraria lobata. A 2024 study by Bai et al. revealed that puerarin alleviates renal inflammatory damage by antagonizing TLR4/MyD88-dependent NF-κB p65 and JNK/FoxO1 activation, thereby inhibiting macrophage polarization toward the M1 phenotype [99].

#### 3.2.2. Novel Biomaterials and Cell Therapy Strategies

Recent advances in nanomedicine have demonstrated groundbreaking strategies for macrophage-targeted therapies through bioinspired nanoparticle engineering [104]. These biocompatible nanoparticles are designed to mimic endogenous biological entities, thereby optimizing their immunocompatibility and tissue-specific targeting capabilities. Notably, reactive oxygen species (ROS)-responsive nanoplatforms enable spatiotemporally controlled drug release in inflammatory microenvironments, where elevated ROS levels trigger the selective liberation of anti-inflammatory payloads. Such stimuli-responsive delivery systems significantly reduce systemic toxicity while enhancing therapeutic efficacy against renal macrophage-mediated pathologies [105,106]. Furthermore, molecular and cellular reprogramming approaches represent frontier interventions for modulating macrophage dynamics in nephrolithiasis. These cutting-edge methodologies harness molecular biology tools to precisely regulate macrophage polarization states and functional phenotypes, offering novel disease-modifying potential.

In 2022, Liu et al. pioneered the synthesis of porous nanorod-structured cerium dioxide nanoparticles (CNPs), which facilitated reversible redox cycling between Ce^3+^ and Ce^4+^. This mechanism effectively ameliorated oxalate-induced oxidative damage in renal tubular epithelial cells, reduced CaOx (CaOx) crystal adhesion to the tubular epithelium, and suppressed CaOx crystallization through enhanced scavenging of reactive oxygen species (ROS) [100]. In 2024, the same research team developed MOF-818 nanozymes, which exhibited dual catalase (CAT)-like and superoxide dismutase (SOD)-like enzymatic activities. These nanozymes modulated the renal inflammatory microenvironment by promoting macrophage polarization from pro-inflammatory M1 to anti-inflammatory M2 phenotypes, thereby attenuating local inflammatory responses. Consequently, this intervention improved renal function parameters (e.g., serum creatinine and urea levels) and significantly decreased renal CaOx crystal deposition in rat models of nephrolithiasis [101]. Most recently, in 2025, Tang et al. engineered a biomimetic nanoparticle system (KM@M@M) comprising hollow mesoporous manganese dioxide (h-MnO_2_) cores encapsulated with macrophage membranes. This system incorporated Kim-1 targeting peptides to achieve site-specific delivery to injured tubules. The nanoparticles co-loaded NLRP3 inflammasome inhibitor MCC950 and h-MnO_2_, synergistically suppressing ROS overproduction, maintaining mitochondrial membrane potential stability, and inhibiting HK-2 cell pyroptosis. In murine models, this strategy markedly reduced CaOx crystal burden and mitigated oxalate-associated renal tubular injury, demonstrating potent therapeutic efficacy against crystal-induced nephropathy [102].

Furthermore, miRNA and epigenetic regulatory factors can be manipulated to regulate the macrophage function and polarization in nephrolithiasis. Specific miRNAs have been proven to modulate the inflammatory response in macrophages, and targeting these miRNAs can change the M1/M2 balance [107]. For example, miR-23 has been demonstrated to reduce crystal deposition and M1 macrophage polarization in the kidneys [45]. In addition, gene editing technologies, such as CRISPR, provide precise methods for regulating macrophage function. CRISPR can be employed to target genes involved in macrophage polarization, permitting specific alterations in macrophage phenotypes. This approach holds significant potential for treating nephrolithiasis by steering macrophages toward a more protective and anti-inflammatory state.

## 4. Future Directions of Nephrolithiasis Research

### 4.1. Novel Technologies and Multi-Omics Integration

#### 4.1.1. Single-Cell and Spatial Omics for Deciphering Macrophage Heterogeneity

Single-cell RNA sequencing (scRNA-seq) has revolutionized our capacity to dissect macrophage population heterogeneity within the nephrolithiasis microenvironment, enabling high-resolution characterization of transcriptional profiles at the individual-cell resolution [108]. When integrated with spatially resolved transcriptomic platforms like 10x Visium, this approach provides multidimensional insights into the spatiotemporal coordination between macrophage polarization and epithelial pathophysiology. A paradigm emerges from silicosis research where spatial omics has mapped macrophage–endothelial crosstalk through colocalization analysis, revealing that pro-inflammatory macrophages impair vascular barrier integrity via MMP12-mediated degradation of neighboring endothelial junctions [109].

#### 4.1.2. Integrative Metabolomics–Immune Microenvironment Profiling

Metabolic reprogramming serves as a central driver of macrophage polarization, with M1 macrophages exhibiting glycolytic dominance while M2 counterparts preferentially engage oxidative phosphorylation [110]. Unveiling the specific enzymes and metabolites involved in these metabolic shifts can provide profound insights into the regulation of macrophage polarization within the nephrolithiasis microenvironment. Emerging evidence indicates pentose phosphate pathway (PPP) activation across various glomerulonephritis models, closely related to the expression of intrarenal macrophage markers, reduced renal function, and increased cytokine production. However, the specific mechanisms still need further exploration [111].

Concurrently, macrophage metabolic reprogramming establishes critical immunometabolic crosstalk bridging microenvironmental dynamics and disease progression. Specific metabolites function as signaling mediators, orchestrating macrophage effector functions through direct pathway modulation or epigenetic remodeling. Pioneering work by Ye’s team at Fudan University revealed that the Immune-responsive gene 1 (IRG1)–itaconate axis drives macrophage immunosuppressive reprogramming in tumor microenvironments, subsequently impairing CD8^+^ T-cell recruitment [112]. Furthermore, the secretion of interleukin 10 (IL-10) and transforming growth factor-β (TGFβ) exerts an inhibitory effect on the function of CD8^+^ T cells through both direct and indirect mechanisms, while CCL22 and TGFβ recruit and promote differentiation of regulatory T cells [113]. These studies underscore the therapeutic potential of integrated immunometabolic profiling in nephrolithiasis, particularly in targeting metabolite-mediated immune checkpoint mechanisms.

### 4.2. Precision Therapeutic Targeting of the Immune Microenvironment

#### 4.2.1. Macrophage-Targeted Nanodelivery Systems

In light of the physicochemical characteristics of the inflammatory microenvironment (e.g., acidic pH, elevated ROS levels), intelligent responsive nanoparticles, such as ROS-sensitive liposomes and pH-responsive exosomes, can be designed to precisely deliver polarization-regulating molecules, including miRNA, interleukin 4 (IL-4), and interleukin-10 (IL-10), to macrophages. Notably, advancements in oncology nanomedicine provide valuable insights—preclinical studies in lung cancer models have revealed that Au@PG nanoparticles can effectively reprogram tumor-associated macrophages (TAMs) by converting anti-inflammatory M2-phenotype TAMs into pro-inflammatory macrophages [114]. This paradigm-shifting approach holds particular promise for nephrolithiasis management, where analogous nanoparticle-mediated macrophage polarization could potentially disrupt the self-perpetuating cycle of crystal deposition and inflammatory damage in renal tissues.

#### 4.2.2. Epigenetic Modulation of Macrophage Reprogramming

Epigenetic modifications—particularly histone post-translational modifications and DNA methylation—exert profound regulatory effects on macrophage polarization patterns [115] and modulate macrophage functionality in nephrolithiasis pathogenesis. During the development and differentiation of immune cells, histone modification, functioning as an epigenetic mechanism that governs gene expression and cell fate, plays a pivotal role. An elevation in the degree of histone acetylation induced by pathogen-associated molecular patterns (PAMPs) can rapidly activate pro-inflammatory genes such as TNF-α, IL-6, and IL-1β, thereby efficiently triggering an immune response. Conversely, histone deacetylases (HDACs) mediate acetylation erasure to terminate transcriptional activation, thereby attenuating inflammatory responses and preventing excessive inflammatory cascades [94]. Importantly, preclinical studies in chronic kidney disease models demonstrate that HDAC3 overexpression promotes macrophage pro-inflammatory polarization through Lys-122 deacetylation of the NF-κB p65 subunit [116]. These findings suggest potential epigenetic vulnerabilities in nephrolithiasis-associated macrophages, though mechanistic insights into histone modification networks during crystal-induced nephropathy await therapeutic exploitation.

#### 4.2.3. Genetically Engineered Macrophage Reprogramming via Precision Editing

Chimeric antigen receptor macrophage (CAR-M) therapy represents a paradigm shift in cellular immunotherapy, utilizing genetic editing to modify macrophages. This modification enables macrophages to express chimeric receptors specific for target antigens, thereby enhancing their ability to recognize and phagocytose pathogens and their immunomodulatory functions. CAR-Ms can be derived from various sources, including peripheral blood, induced pluripotent stem cells (iPSCs), and the human leukemia monocytic cell line (THP-1) [117]. Phase I clinical data published in Nature Medicine in 2025 (NCT04660929) demonstrated that CAR-Ms can effectively migrate to lesions in solid tumors and activate coordinated CD8+ T-cell-mediated immune surveillance [103]. Klichinsky et al. found that CAR-Ms express pro-inflammatory cytokines and chemokines, polarizing them into pro-inflammatory M1-type macrophages. They can also convert bystander M2-type macrophages into M1-type, thereby enhancing anti-tumor immune responses [118]. These findings suggest that CAR-Ms may enhance anti-inflammatory functions and crystal clearance in the renal stone microenvironment through similar mechanisms. At a purely conceptual level, CAR-Ms may enhance anti-inflammatory functions and crystal clearance in the renal stone microenvironment through analogous mechanisms. Future research could enhance macrophage phagocytosis, immunomodulatory precision, and microenvironment remodeling through two synergistic mechanisms: (1) pathogen recognition optimization: CAR-M constructs targeting CaOx crystal-associated proteins (e.g., osteopontin (OPN), CD44 hyaluronan receptor) could ultra-sensitively detect Randall plaque precursors and degrade them in lysosomes; (2) polarization control: engineered CAR-Ms could be programmed to secrete immunomodulatory cytokines (IL-10, TGF-β) or activate PPARγ-mediated transcriptional networks, thereby enhancing M2-like reparative phenotypes and breaking the stone–inflammation–stone vicious cycle.

However, before gene editing technologies can be widely applied in the clinical treatment of nephrolithiasis, ethical considerations and potential off-target effects must be carefully evaluated. Further research is needed to optimize delivery methods for CAR-Ms and minimize the risk of unintended consequences. Despite these challenges, gene editing technologies hold great promise for developing highly targeted and effective therapies for nephrolithiasis by modulating macrophage function.

## 5. Conclusions and Future Perspectives

The new findings about how macrophages change in nephrolithiasis have changed how we treat it. This review shows how different types of macrophages affect CaOx stones. Pro-inflammatory macrophages exacerbate pathological progression by causing too much inflammation and kidney damage through harmful substances like ROS and NLRP3. However, anti-inflammatory macrophages help protect the kidneys by reducing inflammation and cleaning up crystals. New treatments like PPAR-γ drugs, CCR2 blockers, and special nanoparticles show promise for fixing immune problems in stone formation. Also, tools like HDAC inhibitors and CRISPR gene editing now allow more precise control of macrophage behavior. Significant challenges remain. Different patients’ immune systems react differently, and kidney cells/fibroblasts interact with immune cells in complicated ways. Also, new treatments like exosome therapy and nanozymes need long-term safety checks through extended human studies.

## Figures and Tables

**Table 1:** Overview of recent studies on macrophage polarization regulation in nephrolithiasis via chemokine/cytokine and key signaling pathways.

Therapeutic Strategy	Specific Approach	Mechanism of Action	Key Molecules/Targets	Experimental Models	Primary Outcomes	References
Chemokine/Cytokine Regulation	CCR2 Antagonists	Inhibit CCL2/CCR2 axis, reduce M1 polarization, attenuate oxidative stress and inflammation	CCL2, CCR2, TNF-α, IL-6	Glyoxylic acid mouse model/THP-1 cells, HK-2 cells	Reduced M1 polarization, tubular injury, and crystal deposition	[42]
	Recombinant Human CSF-1 Therapy	Enhance M2 polarization, improve phagocytic function	CSF-1/CSF1R signaling pathway	Hyperoxaluric mouse model/BMDMs, RTCs	Increased M2 markers, decreased renal crystals	[43]
	IL-10 Treatment	Activate STAT3 signaling to promote M2 polarization	IL-10, STAT3	High-oxalate-diet human/THP-1 cells	Reversed M1 polarization, reduced crystal deposition and inflammation	[44]
Key Signaling Pathway Targeting	PPAR-γ Agonists (e.g., Pioglitazone)	Bind PPAR-γ to upregulate *miR-23*, suppress IRF1/Pknox1 and shift M1→M2 polarization	PPAR-γ, *miR-23*, IRF1, Pknox1	Glyoxylic acid mouse model/BMDMs	Reduced M1-dominant inflammation, decreased crystal formation	[45]
	NF-κB–NLRP3–IL-1β Feed-Forward Cascade Inhibitors (e.g., FKBP5 Deficiency, NLRP3 Deficiency)	Suppress NF-κB signaling to inhibit M1 polarization, reduce TNF-α/IL-1β secretion	NF-κB, FKBP5, NLRP3, TNF-α, IL-1β	Glyoxylic acid mouse model/BMDMs	Attenuated M1 activation, crystal aggregation, and tubular injury	[46,47]
	Notch Signaling Inhibitors (Sirt1 Overexpression)	Inhibit Notch pathway to promote M2 polarization, reduce renal epithelial cell apoptosis	Notch, Sirt1, TLR4	Glyoxylic acid mouse model/BMDMs, TECs	Enhanced M2 phenotype, reduced crystal deposition and renal fibrosis	[48]

**Table 2:** Recent studies on epigenetic regulation of macrophage polarization in nephrolithiasis.

Therapeutic Strategy	Specific Approach	Mechanism of Action	Key Molecules/Targets	Experimental Models	Primary Outcomes	References
Epigenetic Histone Reprogramming	Histone methyltransferases	Suppress inflammatory cytokine gene transcription or control lipid gene synthesis through histone methylation modifications (e.g., Setdb2, DOTL1)	Setdb2, H3K9me3, NF-κB, DOT1L, H3K79me, Srebf1/2	Diabetic (db/db) mouse model/BMDMs	Shifted macrophages to a reparative phenotype, reduced inflammation	[73,74]
	HDACi, SIRTs	Enhance histone acetylation, promote M2 polarization (e.g., TSA SIRT1/2/3/5/6)	HDAC5, SIRT1/3, NOTCH, FOXO1	Sodium oxalate mouse model/TCMK-1 cells	Reduced M1 polarization, improved M2 reparative phenotype, decreased fibrosis	[75,76]
	ACSS2 inhibitor	Lower the level of histone H3 lysine 9 crotonylation (H3K9cr) to reduce IL-1β-dependent macrophage activation	ACSS2, H3K9cr, IL-1β	UUO mouse model/RAW264.7 cells, TCMK-1 cells	Reduced M1 polarization, delayed tubular cell senescence and the progression of fibrosis	[77]
CpG Methylation Intervention	DNA methyltransferases	Inhibit DNMT3b, promote M2 polarization via PPARγ1 methylation	DNMT3b, PPARγ1	Hydroxyproline rats model/HK-2 cells	Alleviated oxidative stress, crystal adhesion, and stone formation	[78]

**Table 3:** Recent research findings on anti-inflammatory and antioxidant therapies, nanotechnology-based approaches, biomaterials, and gene editing/cellular therapy strategies targeting macrophage polarization in nephrolithiasis.

Therapeutic Strategy	Specific Approach	Mechanism of Action	Key Molecules/Targets	Experimental Models	Primary Outcomes	References
Anti-Inflammatory and Antioxidant Therapies	Natural Products (e.g., SFN, Puerarin)	Suppress TLR4/NF-κB pathway, reduce M1 polarization, enhance Nrf2-mediated antioxidant activity	TLR4, NF-κB, Nrf2, miR-93-5p	Glyoxylic acid mouse model/BMDMs, TECs	Reduced M1 activation, oxidative stress, and crystal adhesion	[98,99]
Nanotechnology and Biomaterials	ROS-Responsive Nanoparticles (e.g., CNPs, MOF-818)	Scavenge ROS, promote M1-to-M2 polarization, modulate inflammatory microenvironment	ROS, CAT, SOD, NLRP3 inflammasome	Ethylene glycol rat model, nude mice/HK-2 cells, NRK-52E cells, NRK-49F cells, MDCK cells	Reduced ROS, improved renal function, decreased crystal deposition	[100,101]
	Biomimetic Nanoparticles (KM@M@M)	Target injured tubules, suppress NLRP3 inflammasome and ROS production	Kim-1 peptide, NLRP3, MnO_2_	Glyoxylic acid mouse model/RAW 264.7 cells, HK-2 cells	Reduced pyroptosis, crystal burden, and tubular injury	[102]
Gene Editing and Cellular Therapy	CRISPR-Cas9	Target genes involved in M1/M2 polarization (e.g., CSF-1, FKBP5)	/	Glyoxylic acid mouse model	Precisely regulated macrophage phenotype, potential for anti-inflammatory shift	[43,46]
	CAR-M Therapy (Conceptual Adaptation for Nephrolithiasis)	Engineer macrophages to express antigen receptors, enhance M1 polarization or phagocytosis	HER2	Phase I clinical trial for solid tumors	Migrated to lesions in solid tumors and activated coordinated CD8+ T-cell-mediated immune surveillance	[103]

## Data Availability

Not applicable.

## Abbreviations

The following abbreviations are used in this manuscript:

CaOx	Calcium Oxalate
TLR4-NF-κB	Toll-like Receptor 4-Nuclear Factor Kappa B
NLRP3	NOD-like Receptor Family Pyrin Domain-containing Protein 3
PPAR-γ	Peroxisome Proliferator-Activated Receptor Gamma
CCR2	C-C Chemokine Receptor Type 2
CSF-1	Colony-Stimulating Factor 1
STAT6	Signal Transducer and Activator of Transcription 6
HDAC	Histone Deacetylase
NF-κB	Nuclear Factor Kappa B
miR	MicroRNA
CRISPR	Clustered Regularly Interspaced Short Palindromic Repeats
CAR-M	Chimeric Antigen Receptor Macrophage
scRNA-seq	Single-Cell RNA Sequencing
AKI	Acute Kidney Injury
IRG1	Immune-Responsive Gene 1
TGFβ	Transforming Growth Factor Beta
CCL2	Chemokine (C-C Motif) Ligand 2
MCP-1	Monocyte Chemoattractant Protein-1
IL	Interleukin
TNF-α	Tumor Necrosis Factor Alpha
ROS	Reactive Oxygen Species
IFN-γ	Interferon Gamma
LPS	Lipopolysaccharide
IRF1	Interferon Regulatory Factor 1
PKNOX1	PKNOX Family Member 1
CAT	Catalase
SOD	Superoxide Dismutase
